# Preventing common mental health problems in war-affected populations: the role of digital interventions

**DOI:** 10.3389/fdgth.2025.1586030

**Published:** 2025-04-10

**Authors:** Iryna Frankova, Marit Sijbrandij

**Affiliations:** ^1^ Department of Clinical, Neuro- and Developmental Psychology, WHO Collaborating Center for Research and Dissemination of Psychological Interventions, Amsterdam Public Health Institute, Vrije Universiteit Amsterdam, Amsterdam, Netherlands; ^2^ Centrum 45, ARQ National Psychotrauma Centre, Oegstgeest, Netherlands

**Keywords:** digital, mental health, prevention, adversity, war

## 1 Introduction

The full-scale Russian Federation invasion of Ukraine in February 2022 has profoundly impacted the lives of millions, and resulting in numerous losses, including the loss of loved ones, health, possessions, social status, and security (1, 2). In addition, over 5 million Ukrainians are now displaced within the country and over 6 million have sought refuge in the European Union. This causes immense stress, uncertainty and significant mental health challenges among both adults and children (3–5). These stressors contribute to mental health issues such as insomnia, depression, anxiety, and post-traumatic stress symptoms (6). Refugee and migrant populations have been shown to be more susceptible to mental health problems compared to the general population, with estimated prevalence rates of up to 32% for depression, 31% for PTSD, and 11% for anxiety disorders (6). However, the mental health care system in Ukraine is overwhelmed and unable to meet the increased demand especially due staff and recourses shortage (7). In addition, countries hosting many Ukrainian refugees, such as Ukraine's bordering countries, may not be able to provide assistance to all Ukrainians in need of mental health care. Scalable interventions such as digital mental health interventions have the potential to enhance access to mental health care and improve mental health conditions (8). There is evidence that digital interventions are as effective as face-to-face alternatives in direct comparisons (9), and the use of mobile, online and other remote technologies for the treatment and prevention of mental disorders in low- and middle-income countries has been reported (10).

While digital mental health interventions are effectively utilized for treatment, this paper aims to focus on the implementation in the field of prevention. E-mental health tools hold significant potential to address various challenges immediately after the onset of a complex emergency. They offer a scalable, timely, and cost-effective approach to fostering resilience and empowering individuals to cope with stress and trauma and allowing emergency services to address the basic needs of survivors. However, little is known about the implementation and effectiveness of digital mental health tools aiming to prevent common mental health issues in the context of complex emergencies and for population affected by war.

## 2 Different phases of war: tailoring digital interventions for mental health in response to complex disasters and mass trauma

Mass casualties like terror attacks, armed conflicts, or war may push a community through exhausting, recurring phases of anticipation, impact, and adaptation before recovery may begin (11). Individuals affected by armed conflicts experience different needs and challenges at various phases. Digital interventions designed to promote the well-being and mental health of individuals affected by complex emergencies must tailor their approaches according to the different phases of the situation. Below we will present two case examples of digital interventions implemented among war-affected Ukrainian populations in different phases of war, aimed at preventing common mental health issues.

### 2.1 Early phases of war and prevention of common mental health problems

As the war in Ukraine was unfolding, countless civilians were exposed to shelling, bombardments, relocation, loss of lives (2). In the early phases of war, individuals often grapple with profound stress and panic, leading to a range of distressing symptoms. These can include tremors, persistent headaches, and overwhelming fatigue. Many may experience a pervasive sense of helplessness and hopelessness, accompanied by tears and a low mood. Anxiety and fear frequently take hold, resulting in uncontrolled emotions and difficulty sleeping, often marked by distressing nightmares. Irritability and anger may surface, alongside feelings of self-blame, guilt, and shame. Additionally, individuals might struggle with a sense of unreality, numbness, and disorientation (12). Those symptoms are usually a response to the direct or indirect impact of life-threatening traumatic experience. The early phases of a war, particularly during the first months, are marked by chaos, uncertainty and lack of communication (13). This period often involves information deprivation, a lack of communication, and uncertainty regarding the available support, as the support infrastructure is profoundly disrupted.

Hobfoll and colleagues identified five critical components of immediate and midterm psychosocial support in the aftermath of trauma exposure (14). These components include ensuring safety; promoting a sense of calm; preserving an individual's sense of self and community efficacy; facilitating social connectedness; and nurturing hope. These elements are broadly recognized as foundational guidelines for formulating prevention strategies and are integral to the recommendations provided during the “golden hours” of early intervention (within first hours and first month) (15).

Universal prevention, aimed at entire population of country at war, or selective prevention targeting individuals who live in a certain proximity to a front-line early enough (16), are often not stigmatizing since people participate in such interventions because they are part of the high-risk groups. Nevertheless the uptake of many universal and selective prevention interventions is low (17). Below, we present a case example in which a large number of individuals utilized the intervention. The key to this high uptake was its delivery in a digital format.

### 2.2 Chatbot intervention based on the principles of psychological first aid

The “Friend” First Aid Chatbot is a Telegram-based self-help tool that was launched on the second day of the full-scale invasion. This conversational agent employs the principles of Psychological First Aid, that gives a framework for addressing initial distress symptoms and supporting people suffering serious crisis events (18, 19, 20). Non-AI decision-tree chatbot intervention offers exercises, tasks, and recommendations in an interactive way to help users cope with acute stress (Table 1). The chatbot adapts to various environments, providing tailored advice whether users are in life-threatening situations or in safe settings. It also informs users about those interventions that might interfere with the spontaneous recovery (21). Available in Ukrainian, English, and Russian, the chatbot has reached over 100 000 users, demonstrating its scalability (22).

**Table 1:** Characteristics and comparison of two digital mental health tools.

Title of the mental health digital tool	Friend first aid	Doing what matters in times of stress
Logo	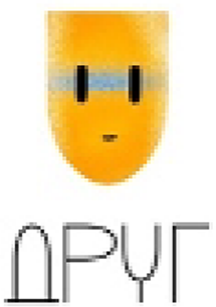	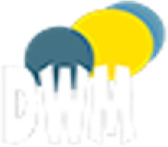
Platform	Messenger	Website
Format	Unguided self-help Telegram-based interventions	Unguided self-help smartphone-based interventions
Therapeutic model or concept	Psychological first aid	Acceptance and commitment therapy, mindfulness-based
For whom	People exposed to traumatic experience within hours—first months	People exposed to adversity
Time invested	Micro-intervention, up to 30 min	5 modules, 20 min each
Languages	Ukrainian, Russian, English	Ukrainian, Russian, English
Access	https://t.me/friend_first_aid_bot	https://dwmatters.eu/

The feasibility of the chatbot was assessed (23, 24). The majority of surveyed users (*n* = 2,207) expressed satisfaction with the intervention, with 45.2% rating it as “Excellent.” A significant portion (39.3%) rated it as “Fair,” while 9.3% rated it as “Bad,” and 6.2% classified it as “Terrible.” Based on written feedback from users (*n* = 1,557), common barriers to engagement with the digital tool included severe mental health issues, technical problems, and a lack of personalization (22). Conversely, facilitators that enhanced satisfaction included social connectedness fostered by the intervention, increased health awareness, and a sense of control over one's own mental health (22). A within-group evaluation (*n* = 817) indicated a medium to large effect size in the reduction of pre-intervention stress levels following the chatbot intervention (25). The results also revealed that parenthood, feelings of safety, and loneliness were predictors of higher stress levels due to exposure to war, highlighting the importance of including advice to caretakers on helping children to better respond and cope with the immediate aftermath of an emergency (25).

### 2.3 Continuous traumatic stress and forced displacement

The ongoing war in Ukraine has already taken an enormous toll on the mental health of many. The continuous traumatic stress is charecterised by increased tension and hypervigilance regarding both current and anticipated threats, as there are no clear boundaries between the onset and cessation of ongoing conflict (26).

We know that people with a migrant background are at increased risk of developing mental health problems such as anxiety, depression and post-traumatic stress related to pre-, peri- and post-migration circumstances (27). Therefore, transdiagnostic approaches targeting prevention of common mental health problems are recommended in reports on interventions for refugee populations (28). Nevertheless, externally displaced people from Ukraine are facing cultural and structural barriers to help seeking, such as mental health related stigma, lack of trust in mental health support, language barriers and lack of mental health professionals (29).

Targeting forcibly displaced persons with digital mental health interventions have a potential to address number of those barriers, such as increasing the reach, bridging language problems and providing help when most needed to individuals difficult to reach. Another advantage of digital self-help interventions is the potential to mitigate stigma associated with seeking mental health support (30). In environments where individuals may experience shame or perceive their needs as less urgent than those of others, digital self-help platforms provide a level of anonymity and self-efficacy.

### 2.4 Doing what matters in times of stress

Doing What Matters in Times of Stress (DWM) is a transdiagnostic, evidence-based digital mental health intervention developed by the World Health Organization (31). An unguided DWM version has been implemented for the Ukrainian population affected by war and forced displacement starting from 2022. DWM serves as a stress management self-help guide that has been adapted into a digital platform. Accessible via smartphone, tablet, or computer, DWM offers five modules based on acceptance and commitment therapy (32). The core concept of DWM is that ongoing attempts to suppress unwanted thoughts and feelings can paradoxically make these problems worse. Instead, the guide emphasizes learning new ways to accommodate difficult thoughts and feelings—primarily through mindfulness approaches—without letting them dominate, while guiding people to take proactive steps towards living in a way that is consistent with their values. DWM is designed for anyone who experiences stress, wherever they live and whatever their circumstances are. A few minutes each day are enough to practice the self-help techniques like grounding, unhooking or acting on one's values. The digital guide can be used alone or with the accompanying audio exercises. DWM has proven effective in reducing psychological distress, anxiety and depression and to improve functioning among healthcare workers in Spain (33) and refugees and migrants in Italy (34).

## 3 Discussion and future directions

The findings presented underscore the critical role of digital mental health interventions in humanitarian contexts, particularly in light of the ongoing armed conflict in Ukraine. The evidence suggests that culturally, linguistically and contextually tailored digital interventions can enhance adherence and efficacy, thus broadening the reach of mental health services in resource-constrained settings (35). As traditional face-to-face interventions become increasingly challenging and time costly to implement, the adaptation and contextualization of existing digital tools emerge as essential strategies for meeting the needs of affected populations as soon as possible (36).

There is still a gap in digital interventions focusing on the unique mental health needs of specific vulnerable groups, such as children. Recent scoping review highlighted limited evidence for the use of digital mental health interventions for children and adolescents affected by war at present (37). As an example of contextualization to a war context, authors suggest including content addressing separation from one or both parents, worry about a parent who is on a battlefield, and ongoing loss and grief. Evidence is emerging about the feasibility and acceptance of the guided chatbot interventions among young people in adversity (38), however, the effectiveness of chatbots remains largely untested (39).

Digital solutions can be integrated with existing efforts in hybrid or blended formats, be offered as stand-alone alternatives with or without guidance. However, the effectiveness of these interventions hinges on adequate infrastructure, including access to technology and reliable internet connectivity.

Another challenge is lack of awareness about the existing effective digital mental health interventions among both forcibly displaced persons and service providers working with that group. Therefore, it is difficult to reach and engage them into those programs (29). Potential solution could be an integration of digital solutions into existing public health infrastructure or humanitarian emergency response, for example along with community-based interventions to create a more holistic approach to mental health care (40, 41).

Thus, digital mental health can complement existing services, it cannot entirely replace the human elements of care, especially in cases requiring specialized support (42). Current evidence suggests that self-guided interventions may be most beneficial for individuals with mild to moderate mental health conditions (43). The limitations of unguided online interventions, particularly for individuals experiencing severe distress, highlight the necessity for further research in this domain.

The recent promising developments in digital mental health and psychosocial support indicate that timely digital interventions have the potential to play a crucial role in preventing the development of chronic mental health problems, such as posttraumatic stress disorder, anxiety and depression following trauma on a large scale, yet the preventive effects of unguided online interventions are still inconclusive and more research is needed (44).

Moreover, there is a growing demand for AI-driven interventions, raising questions about safety and ethical considerations. As digital mental health interventions continue to evolve, it is imperative to conduct rigorous randomized controlled trials to assess the effectiveness of these interventions, particularly in low- and middle-income countries facing humanitarian crises. By embedding prevention programs within existing public health frameworks, we can ensure that digital solutions not only address immediate needs but also contribute to long-term mental health resilience across the lifespan.
